# Development and Validation of an ANN-Based Approach for Temperature-Dependent Equivalent Circuit Modeling of SAW Resonators

**DOI:** 10.3390/mi14050967

**Published:** 2023-04-28

**Authors:** Miloš Radojković, Giovanni Gugliandolo, Mariangela Latino, Zlatica Marinković, Giovanni Crupi, Nicola Donato

**Affiliations:** 1 Faculty of Electronic Engineering, University of Niš, 18000 Niš, Serbia; milos.radojkovic@elfak.rs; 2 Department of Engineering, University of Messina, 98166 Messina, Italy; giovanni.gugliandolo@unime.it (G.G.);; 3 BIOMORF Department, University of Messina, 98125 Messina, Italy

**Keywords:** admittance parameters, artificial neural networks, equivalent circuit model, scattering parameter measurements, surface acoustic wave resonators, resonance, temperature

## Abstract

In this paper, a novel approach is proposed for modeling the temperature-dependent behavior of a surface acoustic wave (SAW) resonator, by using a combination of a lumped-element equivalent circuit model and artificial neural networks (ANNs). More specifically, the temperature dependence of the equivalent circuit parameters/elements (ECPs) is modeled using ANNs, making the equivalent circuit model temperature-dependent. The developed model is validated by using scattering parameter measurements performed on a SAW device with a nominal resonant frequency of 423.22 MHz and under different temperature conditions (i.e., from 0 °C to 100 °C). The extracted ANN-based model can be used for simulation of the SAW resonator RF characteristics in the considered temperature range without the need for further measurements or equivalent circuit extraction procedures. The accuracy of the developed ANN-based model is comparable to that of the original equivalent circuit model.

## 1. Introduction

Surface acoustic wave (SAW) resonators are devices that utilize the properties of piezoelectric materials to convert mechanical vibrations on a solid surface into electrical signals, and vice versa. Due to their high performance and reliability, these devices are widely used in wireless communication systems for the implementation of filters and oscillators [1,2,3]. In recent years, SAW technology has gained significant attention in the scientific community as a highly researched topic, due to its outstanding potential for emerging applications in various fields.

The first SAW device was proposed by White and Voltmer in 1965, who described a method for generating a surface acoustic wave using interdigitated electrodes [4]. However, it was not until the late 1970s that SAW technology began to be commercialized and used in a wide range of electronic devices [5]. These early SAW devices were primarily based on quartz crystal substrates and were utilized in radio communication systems and other high-frequency electronic devices. Over the years, SAW technology has undergone a significant evolution, with the development of new materials and fabrication techniques [1,2,6,7,8]. This has led to the creation of SAW devices with improved performance, increased functionality, and expanded application. Today, SAW technology is used in a wide range of electronic devices and systems, such as 5G wireless communication systems, internet of things (IoT) devices, and wearable technology [9,10,11]. Additionally, the advancements in the technology have opened up new possibilities for SAW technology, such as in the field of sensing applications [7,11,12,13,14].

The working principle of a SAW device is based on the propagation of surface acoustic waves along a piezoelectric substrate. These waves are generated by applying an electric signal to a transducer (e.g., interdigitated electrodes), which creates mechanical vibrations on the surface of the substrate. These vibrations travel along the surface and are received by another transducer, which converts them back into an electric signal. The high stability, high quality factor (Q-factor), and low power consumption of SAW devices make them particularly attractive for use in sensing applications. They can be used to develop a wide range of sensors, including temperature, pressure, and chemical sensors [11,12,13,14,15,16,17]. These sensors are based on the principle of measuring the change in the SAW device resonant frequency caused by changes in the physical quantity being measured. For example, chemical sensors based on SAW devices have been developed for the detection of a wide range of chemical compounds, including gases, liquids, and biomolecules [11,12,14,18,19]. They are very attractive because of their high sensitivity and selectivity, as well as their ability to operate in harsh environments. Additionally, they can be integrated with other devices, such as wireless communication systems, making them a suitable option for use in IoT applications [9,16]. They can also be used in the continuous monitoring of different chemical compounds, making them suitable for applications such as environmental monitoring, industrial process control, and medical diagnostics [11,19,20,21].

Modeling procedures are an important aspect of the design and optimization of SAW devices. Over time, a variety of models have been developed to accurately describe the behavior of SAW resonators, including equivalent circuit models [22], finite elements method (FEM) based models [23,24], and artificial neural networks (ANNs) [25]. Each of these models has its own advantages and limitations.

Equivalent circuit models are based on the electrical representation of the SAW resonator, and they can accurately predict the device resonant frequency and Q-factor. However, the parameter extraction process can be challenging, particularly when dealing with high-frequency devices. Additionally, equivalent circuit models are, typically, valid only for a specific operating condition, such as a specific temperature, pressure, or humidity, making it difficult to accurately predict the behavior of the SAW device under different operating conditions. This represents a major limitation when using an equivalent circuit model.

Unlike equivalent circuit models, finite element models can provide detailed information about the mechanical and piezoelectric properties of the SAW device, which might be very important in some applications, such as sensing. However, they are often computationally expensive, requiring high computational resources.

ANNs can also be used to model SAW resonators. This modeling approach has the advantage of being able to model complex systems, which can be considered as black box systems. In particular, ANNs can learn the relationship between the input and output parameters of the SAW device and can be used, for instance, to predict the resonant frequency and Q-factor of the SAW device at a specific operating condition. Furthermore, they can be trained to generalize the device’s behavior in different operating conditions (e.g., at different temperature or relative humidity levels). For example, ANNs can be used to model the temperature-dependent behavior of SAW devices. This is an important aspect of SAW design and optimization, as the temperature-dependent behavior can affect their performance and stability. As the temperature of the SAW device changes, its resonant frequency and Q-factor are affected [26]. SAW devices are often used in harsh environments, such as industrial or automotive applications, where they are exposed to extreme temperature changes [27]. For this reason, understanding the temperature-dependent behavior of SAW devices under these conditions is crucial for ensuring their reliability and longevity. Furthermore, SAW devices are increasingly being used in IoT applications. Their temperature-dependent behavior can significantly impact the performance of the overall system and this must be taken into account in the design and optimization of such systems.

The present contribution focuses on the modeling of a commercial SAW resonator over a temperature range between 0 °C and 100 °C by using an ANN approach for the determination of the lumped-element values of an equivalent circuit model. The device is a two-port packaged SAW characterized by a nominal resonant frequency of 423.22 MHz. Unlike the equivalent circuit model [26], where for each temperature it is necessary to repeat measurements of the S-parameters and extraction of the ECPs, in the present approach the model has ANNs assigned to the equivalent circuit model, aimed at predicting the ECPs for any temperature in the considered range, without the need for additional measurements and optimizations. In that way, a temperature-dependent equivalent circuit model is built. Firstly, the lumped-element values of the equivalent circuit model for the SAW under test are analytically extracted from the scattering (S-) parameter measurements in the entire range of the investigated temperature [26]. Next, an ANN is trained by using the extracted values with the aim of generalizing the SAW behavior and calculating the lumped-element values at different operating conditions (i.e., at different temperatures). The developed model is referred to as the ECP ANN, as it is based on using a standard equivalent circuit model that is empowered by using ANNs. It is worth noting that the equivalent circuit model employed in this work has been already tested and successfully validated in a wide temperature range [22,28,29].

The rest of the article is organized as follows. Section 2 briefly describes the SAW device employed in this work as well as the artificial neural networks chosen as the method exploited for modeling. In Section 3, the proposed ECP ANN modeling approach is described, while the main results are reported and discussed in Section 4. Finally, the concluding remarks are given in the last section.

## 2. Materials and Methods

This section is divided into two parts. The first subsection is devoted to the description of the tested SAW device, whereas the second subsection presents a short introduction of artificial neural networks. 

### 2.1. Modeled SAW Resonator

The resonator under examination is a SAW device in a TO-39 package (from Murata, Kyoto, Japan) with the manufacturer code SAR423.2MDA30x80 [30]. The designation “SAR” stands for SAW resonator [31], “423.2M” indicates that the resonant frequency is 423.2 MHz, “DA” signifies that the SAW has two ports, “3” indicates that it uses the TO39-3 leaded package, “0” implies that the specification designator is standard, “x” represents the use of a quartz substrate, and “80” denotes a frequency tolerance of 80 kHz.

The resonator was characterized based on its admittance (Y-) parameters at various temperatures, ranging from 0 °C to 100 °C. This was carried out by using a vector network analyzer (VNA) to measure the four complex S-parameters, which were then transformed into the corresponding four complex Y-parameters [32]. The use of admittance parameters was chosen for this investigation as they provide a more convenient representation for easily determining the values of the equivalent circuit elements. The temperature-dependent measurements were performed using a temperature control system. In particular, the SAW resonator under examination was placed in contact with a Peltier cell equipped with a Pt100 thermoresistance. This setup enabled temperature actuation and control during the measurement process. The Agilent E3631A power supply provided the necessary voltage to the cell, while the Agilent 34401A digital multimeter was used to measure the thermistor resistance as a feedback signal for the temperature control. The electrical characterization of the SAW was carried out measuring the S-parameters with an Agilent 8753ES VNA in the frequency range going from 422.74 MHz to 423.74 MHz.

All of the instruments used in this study were connected to a desktop PC through the IEEE 488.2 protocol. The PC served as a central control unit for the measurement setup, allowing for the control of each instrument and the real-time data acquisition and post-processing analysis through the use of custom-made software. A more detailed description of the measurement setup can be found in [26].

A full two-port calibration procedure was carried out on the VNA using a specially designed calibration kit consisting of short-open-load-through (SOLT) standards [33]. This procedure is essential as it aligns the measurement reference planes with the SAW pins, effectively eliminating the contribution of cables and connectors in the measurement results.

The measurement system used in this study was capable of conducting measurements above and below room temperature with a maximum temperature difference of 68 °C between the two plates of the Peltier cell. To prevent overheating of the Peltier cell hot plate during the measurements below room temperature and to ensure the longevity of the cell, a heat sink coupled with a fan was incorporated into the Peltier cell setup. This cooling system not only protected the cell, but also improved its performance, enabling measurements to be taken across a wider temperature range. The temperature of the cell was regulated through a closed-loop proportional–integral–derivative (PID) control system. The feedback signal was provided by the Pt100 thermoresistor, which was placed alongside the SAW under test in contact with the Peltier cell. Specially made software was used for implementing the virtual PID control unit, measuring the S-parameters with the VNA, and converting them into the corresponding Y-parameters.

### 2.2. Artificial Neural Networks

Artificial neural networks are structures inspired by the natural nervous system. Similarly to natural neural systems, ANNs consist of interconnected units called neurons [34]. Neurons process information coming from stimuli originating from the outside environment or from connected neurons. The ANNs exploited in this work are multilayer perceptron (MLP) neural networks. In MLP ANNs, neurons are grouped into layers. Within a layer, the neurons are not mutually interconnected, but all the neurons from one layer are connected to all the neurons of the next layer. In terms of ANN theory, these layers are named fully connected layers. Each connection is weighted, i.e., the value at the output of a neuron is multiplied by the corresponding connection weights before passing to the neurons of the next layer. A multilayer perceptron ANN receives input information from the environment through the neurons of the input layer. Then, the information is passed to the hidden layers and, finally, to the output layer. The responses of the neurons in the output layer are the outputs of the neural network. An activation (transfer) function is assigned to each neuron and it should be properly selected from several available possible functions (e.g., sigmoid functions such as the logistic sigmoid function, hyperbolic tangent function, linear functions such as the pure line function or rectified linear unit (ReLU) function, etc.). For instance, the activation functions of the hidden layer neurons are usually sigmoid functions, whereas the output neurons have linear functions. All the neurons from a layer have the same activation function but with different function thresholds. Therefore, the output of a layer is determined by applying the transfer function to the sum of the weighted outputs of neurons from the previous layer with the threshold value added. In the same way that a natural neural system learns from experience by making new connections among the neurons and/or strengthening/weakening the synapses between the already connected neurons, artificial neural networks learn the input–output dependences by adjusting the values of the connection weights and thresholds of the activation functions. This is an optimization process that is referred to as ANN training. It is performed by applying appropriate optimization procedures (i.e., by executing training algorithms, such as the backpropagation algorithm, Levenberg-Marquardt algorithm, quasi-Newton algorithm, etc.) [35]. During ANN training, the weights and thresholds are optimized with the aim of making the ANN response as close as possible to the desired outputs. The training of MLP ANNs is classified as a supervised method, meaning that the desired ANN outputs for several combinations of the values of the ANN inputs are known and used in the training procedure. The set of known input/output combinations is called a training set. Therefore, an ANN learns the relationship between the inputs and outputs. It is important that the set of data used for the training set should be chosen in a such way that the ANN input space is properly covered. A feature of ANNs that makes them highly suitable for developing models to resolve engineering problems is that they can provide a correct response not only for the input–output combinations from the training samples, but also for input combinations that are not present in the training set. To ensure this occurs, the ANN response should be validated by comparing the ANN responses and the desired values not only for the input combinations from the training set, but also for the input combinations from a test set different from the training one. After the training is completed, the parameters of the ANN will not be changed any more, and the ANN is ready to be further used.

As far as the field of RF and microwaves is concerned, ANNs have become one of well-established technologies for the modeling, simulation, and optimization of microwave circuits and devices, a process that began more than two decades ago and is continuing to develop [35,36,37]. They have been applied to a variety of passive and active devices, starting with microstrip lines, microwave filters, field effect transistors (FETs), and printed antennas, to novel advanced devices such as GaN high electron mobility transistors (HEMTs), micro-electro/mechanical (MEMS) devices, sensors, etc. [35,36,37,38,39,40,41,42,43,44,45]. The vast majority of applications are based on simple black box models, in which ANNs are used to establish directly the relationship between the input and output datasets. However, there are significant numbers of applications in which ANNs are combined with external models (these combined models are also called hybrid models or surrogate models), i.e., where knowledge about the problem being modeled is incorporated into the model. The most exploited knowledge-based neural approaches include the space mapping (SM) modeling approach and prior knowledge input (PKI) modeling approach [35]. Depending on the application, knowledge-based models are applied to extend the model’s validity, to improve the accuracy of the model (either of a neural model or of an existing model), to reduce the number of samples needed for training the ANNs of which the model is composed, etc.

Most of the common models of devices and circuits used in standard microwave circuit simulators do not include temperature dependence, i.e., they are valid at only one temperature and for any other temperature the model has to be re-extracted. On the other hand, electromagnetic (EM) simulators or multiphysics simulators, which support temperature-dependent modeling, are often based on complex calculations, thereby making the simulations and optimizations of the circuits containing the devices whose characteristics are temperature-dependent time-consuming. There have been many attempts to develop alternative models and modeling approaches dealing with the temperature dependence of the behavior of different microwave circuits and devices [46,47,48,49,50,51,52,53]. Among them, ANNs have been proved to be a very efficient solution to develop such models, either purely based on ANNs or combined with existing models. As will be described in the next section, the model proposed in this work exploits the space mapping neural modeling approach to introduce temperature dependence to the equivalent circuit model of the SAW resonator.

## 3. Proposed ECP ANN Modeling Approach

**Figure 1:**
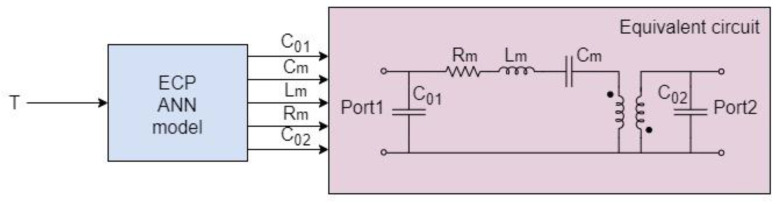
Proposed temperature-dependent ECP ANN SAW model based on the equivalent circuit.

In the proposed approach, the temperature dependence of the RF characteristics of the modeled SAW resonator is implemented in the equivalent circuit by associating a temperature-dependent neural model of the equivalent circuit parameters/elements (ECPs) with the SAW equivalent circuit model (see Figure 1). The ECP temperature-dependent neural model consists of ANN(s) trained to model the temperature dependence of the ECPs. Mathematical expressions corresponding to the developed ECP ANN model are assigned to the equivalent circuit schematic through blocks dealing with expressions and variables, resulting in the temperature-dependent equivalent circuit. Once the model is developed, the RF behavior of the resonator can be determined for any temperature from the considered temperature range, without the need for any additional measurement or modification in the developed model. Regarding the type of neural model, the ECP ANN model belongs to the group of space mapping neural models, as the ANNs are used to map the input space of temperature to the input space of the equivalent circuit model, which are the ECPs.

**Figure 2:**
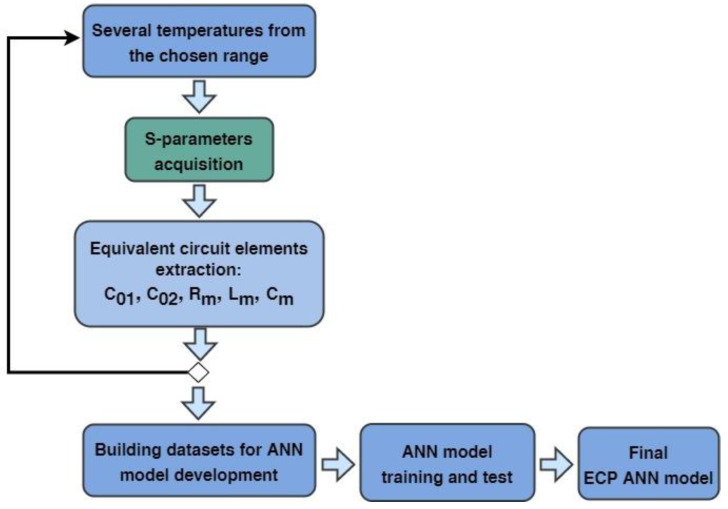
Schematic illustration of the proposed procedure for ECP ANN model development.

The procedure for the model development is as follows (see Figure 2). At the beginning, it is necessary to acquire the S-parameter measurements for several temperatures selected from within the temperature range that is being considered for the model development. For each selected temperature, the equivalent circuit elements are extracted. This task can be accomplished by using analytical or optimization procedures, or even a combination of the two. Then, the extracted data are used to make datasets for developing ANNs aimed at modeling temperature dependence. The development of the ANN-based model will be described in detail in the next section. After the ANN model is developed, the mathematical expressions corresponding to the ANN model are assigned to the schematic of the equivalent circuit in a circuit simulator, thereby composing the final temperature-dependent equivalent circuit model of the studied resonator.

**Figure 3:**
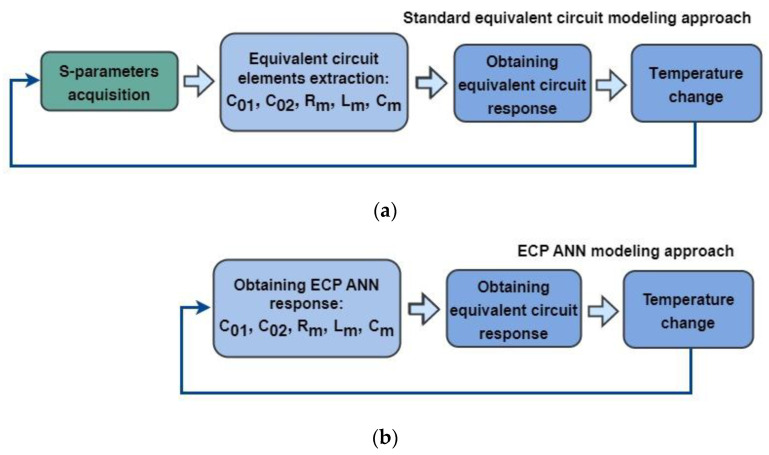
Schematic illustration of the model exploitation: (**a**) Standard equivalent circuit modeling approach and (**b**) ECP ANN modeling approach.

Regarding the model exploitation, a comparison of this model and the standard equivalent circuit model is illustrated in Figure 3. As mentioned in the previous paragraphs, the standard equivalent circuit model needs the acquisition of the S-parameters and ECP extraction procedures for each temperature of interest (see Figure 3a), whereas in the case of the proposed ECP ANN approach, the ECPs for any temperature are determined by obtaining the ANN model response, i.e., by calculating the outputs of the set of mathematical expressions describing the ECP ANN model, for the considered temperature (see Figure 3b). In this way, the simulation becomes more efficient and the need for further measurements and additional model extraction is avoided. It should be noted that the range of temperatures at which the model is valid is determined by the range of temperatures used in the training set.

## 4. Experimental Results and Discussion

The proposed model was verified using the SAW resonator described in Section 2. For this device, the S-parameters were measured for six temperatures in the range from 0 °C to 100 °C, with steps of 20 °C. For each of the analyzed temperatures, the elements of the equivalent circuit model illustrated in Figure 1 were determined using the procedure reported in [26], which is based on a combination of the Lorentzian fitting approach [54] and analytical extraction [22]. These data were used for developing the ANN model. The model is based on multilayer ANNs. More specifically, the model is a multilayer perceptron ANN that has one neuron, corresponding to the temperature, in the input layer and five neurons, corresponding to the five modeled ECPs (i.e., *C*_01_, *R*_m_, *C*_m_, *L*_m_, and *C*_02_), in the output layer. The considered ANNs have one or two layers of hidden neurons. The number of hidden neurons was properly determined during the model development. More specifically, several ANNs with different numbers of hidden neurons were trained and compared in terms of training and generalization accuracy. The training accuracy indicates how well the ANNs have learnt the training data, whereas the generalization accuracy indicates the accuracy of the ANN model response for the input values not used for the model development. The ANN having the best compromise between training and generalization accuracy was chosen as the final empowered model, namely the ECP ANN model. The ANNs were trained using the Levenberg Marquardt training algorithm [55], which is widely used for training multilayer perceptron neural networks.

Bearing in mind that the training set consisted of just six samples, corresponding to the six available temperatures used to extract the ECPs, saving any of the samples for the independent test of generalization would remove from the training set important information about the ECP behavior. Therefore, to ensure that the ANN input space is properly covered, all available data were used to train the ANNs. However, training ANNs with a small amount of data may easily result in the ANNs overlearning, resulting in very poor generalization abilities for the input values that differ from the training ones. As all the data were used for the training set and therefore it was not possible to provide a test set independent of the training one, to ensure that the ANNs did not exhibit overlearning, the continuous response of the ANNs versus temperature (i.e., steps of 1 °C or even less) was visually checked for detecting possible overlearning. In the considered case, the best results were achieved with the ANN that with one layer consisting of three hidden neurons with the logistic sigmoid activation function. The input neuron has the unitary activation function, and the output neurons have the linear activation functions.

**Figure 4:**
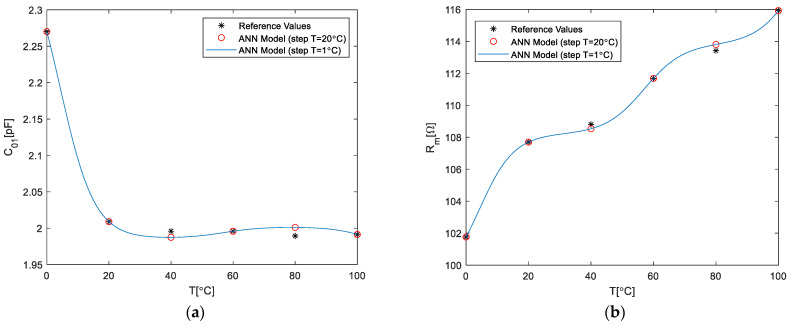

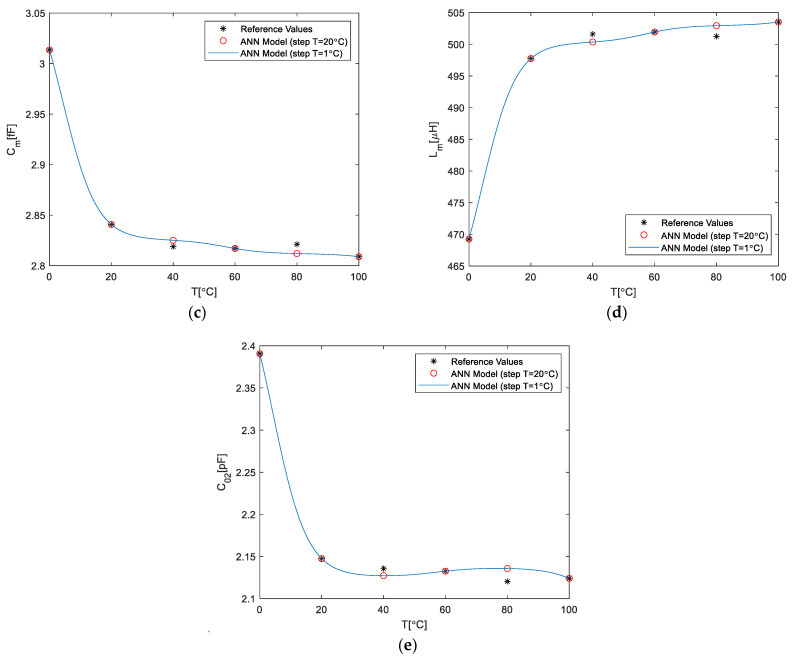
Comparison between ECPs determined by the ECP ANN model (lines with circles) and the ECP reference values (stars) versus temperature: (**a**) *C*_01_, (**b**) *R*_m_, (**c**) *C*_m_, (**d**) *L*_m_, and (**e**) *C*_02._

Figure 4 shows the comparison of the chosen ECP ANN model response with the reference values of the ECPs (stars in the plot), which were used for the training set. In addition to the values of the ANN response at the considered six temperatures (i.e., temperature steps of 20 °C, circles in the plots), the continuous response of the model (i.e., temperature steps of 1 °C, lines in the plots) are shown. It can be observed that the modeled values successfully follow the reference data, thereby indicating that good training accuracy has been obtained; and the continuous response does not exhibit any undesired behavior (i.e., there are no signs of over-fitting), thereby indicating that good generalization has been achieved. Although it might seem that the ECPs at some temperatures exhibit higher deviations, the actual change is considerably small, as confirmed by the relative errors (RE) of the prediction of the ECPs (see Table 1). As can be seen, the relative errors in almost all cases are less than 0.5%, and in the majority of cases are less than 0.001%. Therefore, as the model exhibits both good learning accuracy and good generalization without signs of over-fitting, it was further implemented in the equivalent circuit schematic. 

To implement the developed ECP ANN model in a circuit simulator, expressions that are equivalent to the trained ANN were added to the equivalent circuit in a block dealing with variables and expressions (VAR block). The calculated outputs of the VAR block are the values of the ECPs that are assigned to the corresponding elements of the equivalent circuit. The expressions describing the trained ANN are given in Table 2. It should be noted that, in this particular case, in order to put the neurons into their most sensitive areas, the input and output training data were pre-processed by converting the original ranges to the −1 to 1 range. This means that when the ANN is used, the input should be post-processed, i.e., it should be converted to the −1 to 1 range (the pre-processing block in Table 2) and the outputs should be converted back from the −1 to 1 range to the original range (the post-processing block in Table 2). The pre-processing and post-processing expressions can be given in a more compact form, but for the sake of illustration here the full expressions dealing with the minimum and maximum values of each group of considered parameters (input and output) are shown. There are also blocks corresponding to the hidden layer and output layer, whereas there is not an expression block for the input layer, as its transfer function is *y* = *x*, having only a buffer role. 

**Figure 5:**
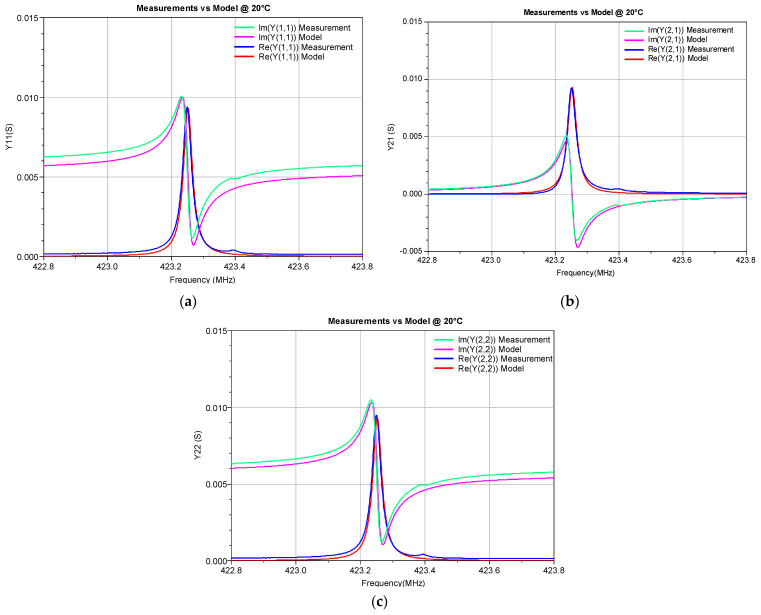
Real and imaginary parts of the Y-parameters of the SAW resonator at 20 °C: (**a**) *Y*_11_, (**b**) *Y*_21_, and (**c**) *Y*_22._ The measurements are compared with the simulations obtained by the proposed ECP ANN model.

**Figure 6:**
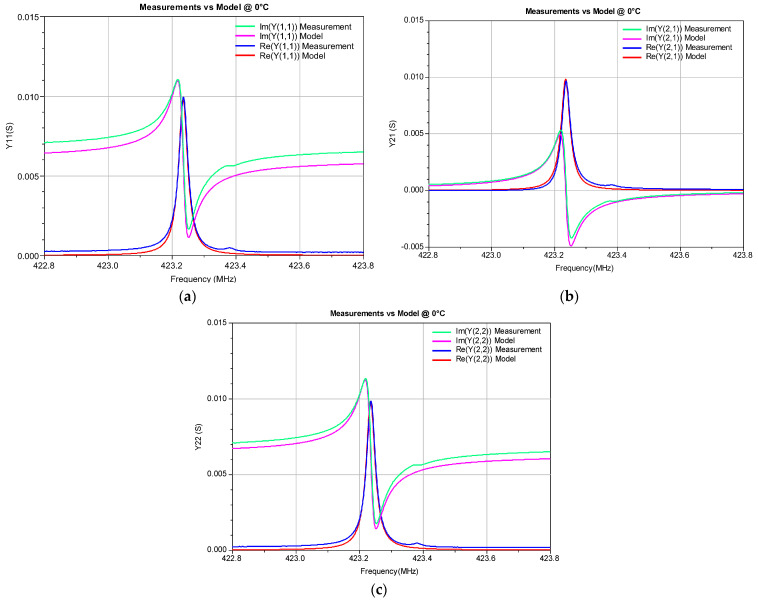
Real and imaginary parts of the Y-parameters of the SAW resonator at 0 °C: (**a**) *Y*_11_, (**b**) *Y*_21_, and (**c**) *Y*_22._ The measurements are compared with the simulations obtained by the proposed ECP ANN model.

**Figure 7:**
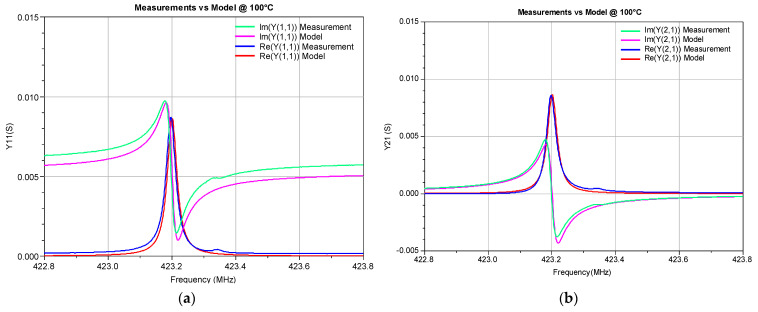

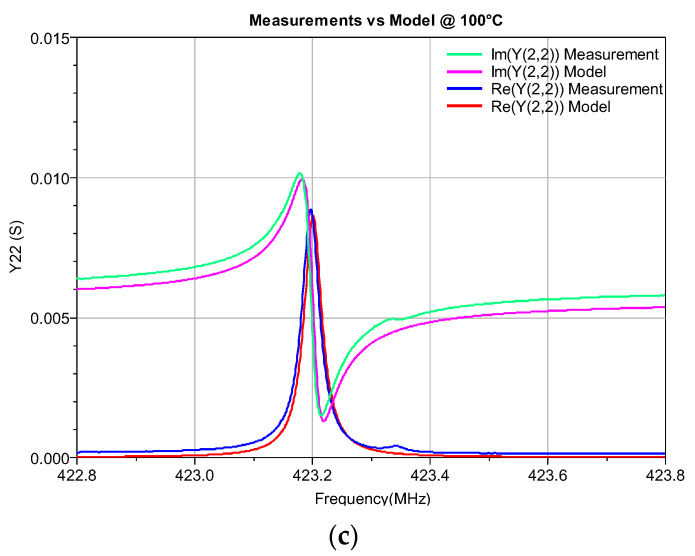
Real and imaginary parts of the Y-parameters of the SAW resonator at 100 °C: (**a**) *Y*_11_, (**b**) *Y*_21_, and (**c**) *Y*_22._ The measurements are compared with the simulations obtained by the proposed ECP ANN model.

**Figure 8:**
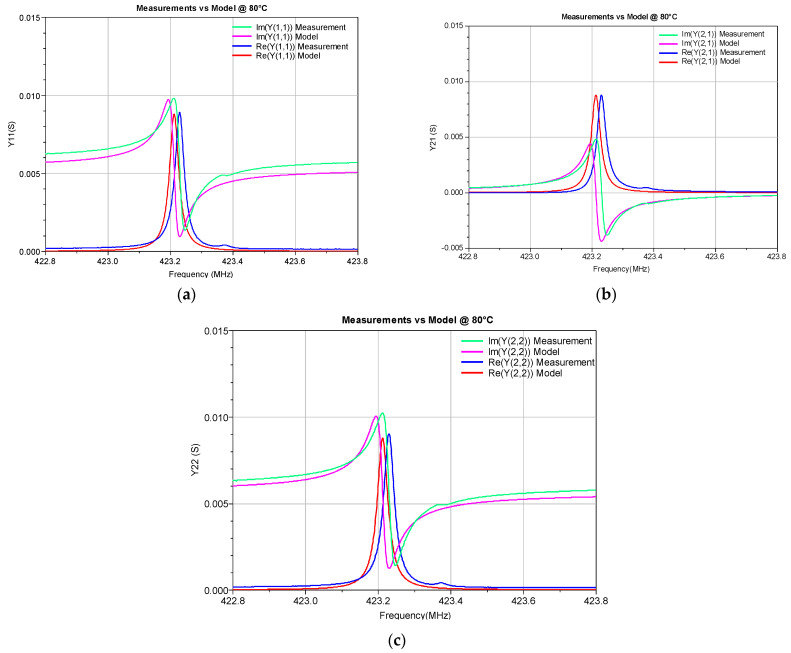
Real and imaginary parts of the Y-parameters of the SAW resonator at 80 °C: (**a**) *Y*_11_, (**b**) *Y*_21_, and (**c**) *Y*_22._ The measurements are compared with the simulations obtained by the proposed ECP ANN model.

To illustrate the accuracy of the developed ECP ANN model, the Y-parameters simulated by this model in a circuit simulator were compared with the corresponding measured values (see Figure 5, Figure 6, Figure 7 and Figure 8). The measured Y-parameters were obtained from the measured S-parameters using the standard S-to-Y conversion formulas [32]. The plots refer to the frequency range in which the measurements were performed. As the device is reciprocal (i.e., *Y*_21_ = *Y*_12_), only the *Y*_11_, *Y*_12_, and *Y*_22_ parameters are shown. Figure 5 refers to room temperature (20 °C) and Figure 6 and Figure 7 refer to the minimum (0 °C) and maximum (100 °C) considered temperatures, respectively. Figure 8 shows the comparison of the measured and simulated Y-parameters for the temperature of 80 °C for which the ECPs deviate the most from their reference values (although deviations of all elements are less than 1%, as already concluded from Table 1). It can be observed that the model provides values that are very close to the measured ones, especially around the resonant frequency *f_r_*. The frequency *f_r_* is the frequency at which Re(*Y*_11_) achieves its maximum amplitude. In Table 3, the resonant frequency determined from the model simulations and the corresponding one determined from the measured data (reference values) are reported. Moreover, the absolute differences of the resonant frequencies obtained by the simulations and measurements (Δ*f_r_*), and the corresponding relative errors (RE), are also given in Table 3.

## 5. Conclusions

ANNs were exploited to model the temperature dependence of the ECPs of an earlier proposed equivalent circuit model of a SAW resonator. The developed approach is referred to as ECP ANN approach. The data used for training the ANN were obtained by an extraction procedure based on the Lorentzian fitting of the device Y-parameters and an analytical modeling technique. Once the ANN is developed and the mathematical expressions are assigned to the equivalent circuit model, the model becomes temperature-dependent and simulations of the SAW resonator RF characteristics can be simulated easily for any temperature selected from the considered temperature range. In the present case, the model is developed for the temperature range spanning 0 °C to 100 °C. The Y-parameters obtained with the proposed model agree very well with the measured data.

It should be mentioned that the accuracy of the developed temperature-dependent equivalent circuit model cannot exceed the accuracy of the original equivalent circuit model. More specifically, the accuracy of the proposed ECP ANN model depends strongly on the accuracy of the standard equivalent circuit model itself, since the ECPs are used for the ANN training, and, of course, on the accuracy of the measured data from which the ECPs are obtained.

## Figures and Tables

**Table 1:** Relative errors (RE) of the ECP prediction in comparison to the reference values at the investigated temperatures.

*T* [°C]	*C*_01_—RE [%]	*R*_m_—RE [%]	*C*_m_—RE [%]	*L*_m_—RE [%]	*C*_02_—RE [%]
0	4 × 10^−4^	7 × 10^−5^	2 × 10^−4^	2 × 10^−4^	3 × 10^−4^
20	1 × 10^−3^	2 × 10^−5^	6 × 10^−4^	5 × 10^−4^	6 × 10^−4^
40	0.42	0.24	0.22	0.25	0.4
60	2 × 10^−3^	4 × 10^−4^	1 × 10^−3^	7 × 10^−4^	7 × 10^−4^
80	0.58	0.34	0.32	0.34	0.72
100	4 × 10^−5^	3 × 10^−6^	2 × 10^−5^	2 × 10^−5^	2 × 10^−5^

**Table 2:** Mathematical expressions describing the ANN modeling ECPs with pre-processing and post-processing expressions included.

%% pre-processing
p1n = 2 × (T + 0)/(100 + 0) − 1
%% hidden layer
h1 = 1/(1 + exp(−(+7.79 × p1n − 9.008)))
h2 = 1/(1 + exp(−(−8.0534 × p1n + 1.129)))
h3 = 1/(1 + exp(−(+8.9945 × p1n + 8.2306)))
%% output layer
o1 = −0.33237 × h1 − 0.11563 × h2 − 2.9694 × h3 + 2.0592
o2 = +1.3054 × h1 − 0.7926 × h2 + 1.3343 × h3 − 0.63149
o3 = −0.12528 × h1 + 0.13354 × h2 − 2.6941 × h3 + 1.7226
o4 = +0.13941 × h1 − 0.15795 × h2 + 2.6537 × h3 − 1.6853
o5 = −0.41034 × h1 − 0.080109 × h2 − 2.8719 × h3 + 1.9927
%% post-processing
C01 = 0.5 × (o1 + 1) × (2.2701 × 10^−12^ − 1.9894 × 10^−12^) + 1.9894 × 10^−12^
Rm = 0.5 × (o2 + 1) × (115.9285 − 101.7618) + 101.7618
Cm = 0.5 × (o3 + 1) × (3.0135 × 10^−16^ − 2.8089 × 10^−16^) + 2.8089 × 10^−16^
Lm = 0.5 × (o4 + 1) × (0.00050351 − 0.00046925) + 0.00046925
C02 = 0.5 × (o5 + 1) × (2.3905 × 10^−12^ − 2.1204 × 10^−12^) + 2.1204 × 10^−12^

**Table 3:** Resonant frequency obtained by the proposed ECP ANN model compared with the reference (measured) values at the investigated temperatures. The absolute and relative errors are reported.

*T* [C]	*fr* [MHz]—Meas	*fr* [MHz]—Model	Δ*fr* [kHz]	*fr*—RE [%]
0	423.237	423.236	1	2 × 10^−4^
20	423.251	423.253	2	4 × 10^−4^
40	423.256	423.316	60	0.01
60	423.249	423.253	4	9 × 10^−4^
80	423.230	423.212	18	4 × 10^−3^
100	423.198	423.201	3	7 × 10^−4^

## Data Availability

Not applicable.
